# Preoperative decision tree model for predicting pulmonary valve-sparing repair in humanitarian pediatric tetralogy of fallot patients

**DOI:** 10.1186/s13019-026-03882-8

**Published:** 2026-03-02

**Authors:** Vitor Mendes, Abdelkhalek Mouloudi, Jalal Jolou, Tomasz Nalecz, Ana Abecasis, Telmo Pereira, Tornike Sologashvili

**Affiliations:** 1 Division of Cardiovascular Surgery, Department of Surgery, Geneva University Hospitals (HUG), Geneva, 1211 Switzerland; 2 Global Health and Tropical Medicine, Institute of Hygiene and Tropical Medicine, NOVA University of Lisbon (IHMT-UNL), Lisbon, Portugal; 3 H&TRC - Health & Technology Research Center, Coimbra Health School, Polytechnic University of Coimbra, Coimbra, Portugal

**Keywords:** Tetralogy of fallot, Pulmonary valve-sparing repair, Decision tree, Humanitarian patients

## Abstract

**Background:**

Pulmonary valve-sparing repair in Tetralogy of Fallot is associated with better long-term outcomes, but its feasibility remains uncertain in pediatric humanitarian patients, who often present late, with complex anatomy and comorbidities.

**Objective:**

To develop a simple and interpretable decision tree model to predict the likelihood of pulmonary valve-sparing repair based on preoperative data in a humanitarian pediatric cohort.

**Methods:**

A post-hoc classification and regression tree analysis was conducted on 115 pediatric humanitarian patients with Tetralogy of Fallot who underwent surgical correction at our center, between 2019 and 2023. Predictor variables included demographic, anthropometric, and anatomical parameters.

**Results:**

The model achieved an overall accuracy of 80.0%, with a sensitivity of 88.2% and specificity of 56.7%. The pulmonary valve annulus diameter (> 0.855 cm) was the primary discriminator for pulmonary valve-sparing repair.

**Conclusion:**

A simple decision tree model using preoperative anatomical variables may support surgical planning in humanitarian contexts. Its interpretability and high sensitivity suggest potential value as a preoperative triage and planning aid.

**Supplementary Information:**

The online version contains supplementary material available at 10.1186/s13019-026-03882-8.

## Introduction

Surgical correction of Tetralogy of Fallot (TOF) is essential for long-term survival and is ideally performed within the first months of life [1]. Pulmonary valve-sparing repair (PV-SR) has been associated with better functional outcomes and reduced long-term pulmonary regurgitation compared to transannular patch repair or right ventricle-to-pulmonary artery conduits (RV-PA conduits) [2, 3].

Pediatric humanitarian patients – children from low-resource countries referred for surgery through international cooperation – often present late, with advanced disease, absent prior palliation, and multiple comorbidities [4, 5]. These factors can limit the feasibility of PV-SR and negatively influence surgical outcomes.

In our previous study, we used multivariate logistic regression, to identify predictors of PV-SR, highlighting the importance of pulmonary valve annulus diameter (measured in both centimeters and Z-score) and body mass index (BMI) [6].

Although the decision to perform PV-SR is ultimately made intraoperatively based on direct anatomical assessment, no validated preoperative tool currently exists to guide surgical planning, particularly in humanitarian patients.

To our knowledge, no existing preoperative model tailored to humanitarian pediatric patients with TOF has been published.

Machine learning algorithms, such as decision trees, can incorporate multiple variables and their interactions while remaining interpretable and practical for clinical use [7]. Their intuitive structure makes them especially valuable in environments where decisions must be made quickly and reproducible.

Building on our existing dataset, this analysis explores the use of a decision tree model based exclusively on preoperative variables to identify candidates for PV-SR.

The aim of this study is to develop a simple, accessible and clinically intuitive tool to support surgical decision-making in pediatric humanitarian patients with TOF, prioritizing preoperative anatomical and clinical variables that are broadly applicable across settings.

## Materials and methods

A post-hoc classification and regression tree (CRT) analysis was conducted on a cohort of 115 pediatric humanitarian patients with TOF, who underwent corrective surgery between 2019 and 2023, as part of a humanitarian surgical collaboration program. All patients originated from low- and middle-income countries (LMICs) and were referred for surgery at our center. The inclusion criteria and clinical outcomes have been previously described [6].

This post-hoc analysis was conducted and reported in accordance with the TRIPOD (Transparent Reporting of a multivariable prediction model for Individual Prognosis Or Diagnosis) statement for the development of multivariable prediction models [8]. Although machine learning terminology is used, the proposed model is a traditional CRT based on fixed splitting rules and prespecified predictors. It does not involve deep learning, representation learning, or adaptative algorithms; therefore, TRIPOD-AI items were considered only where applicable, with TRIPOD remaining the primary reporting framework. The completed TRIPOD checklist is provided as Supplementary Material (Supplementary material- checklist).

Patients who had previously undergone any form of preoperative palliation, including right ventricular outflow tract (RVOT) stenting, were excluded from the study. No patient in the final cohort had undergone RVOT stenting or other palliative interventions.

Pulmonary artery branch diameters (LPA and RPA) were not available in the original dataset and therefore could not be included in the present analysis.

Only patients with complete preoperative data were included in the analysis. Patients with missing preoperative data were excluded.

All procedures were performed by the same congenital cardiac surgery team. The decision to preserve the native pulmonary valve was made intraoperatively after direct inspection of the pulmonary annulus, valve morphology, and RVOT.

The pulmonary annulus and RVOT were systematically assessed using Hegar dilators. The target annular diameter was determined according to the patient’s body surface area (BSA), using our institutional reference chart expected normal annular size. Surgeons selected the dilator corresponding to the theoretical normal annular diameter, with the surgical goal of achieving an RVOT dimension equivalent to a Z-score of approximately 0, while maintaining leaflet mobility and annular integrity.

Immediate postoperative RVOT gradients were assessed intraoperatively using transoesophageal echocardiography following weaning from cardiopulmonary bypass (CPB). At our institution, a maximal instantaneous RVOT gradient of ≤ 30–35 mmHg is considered acceptable following PV-SR. Gradients > 35–40 mmHg prompt reassessment of RVOT anatomy and valve function.

A return to CPB is undertaken in cases of persistent residual RVOT gradient > 35–40 mmHg after hemodynamic optimization, or when inspection or echocardiographic evaluation reveals leaflet restriction, annular distortion, significant right ventricular dysfunction, or persistent subvalvular obstruction. These intraoperative hemodynamic parameters are part of standard clinical practice; however, because they represent intraoperative and postoperative findings rather than preoperative characteristics, they were not included as predictor variables in the CRT model, which was limited to preoperative data.

A PV-SR was pursued when adequate relief of RVOTO could be achieved without excessive commissural stretching or distortion of leaflet geometry. If these conditions were not met, or if residual RVOT gradients remained unacceptable upon intraoperative assessment, a transannular patch repair or RV-PA conduit implantation was performed.

The dependent variable was the binary classification of whether a PV-SR, was performed (yes/no). The cutoff value of 0.855 cm for pulmonary valve annulus diameter was derived from our previously published study using ROC curve analysis with Youden index to optimize sensitivity and specificity, and was applied a priori in this post-hoc CRT analysis [6]. Predictor variables included demographic (country of origin, sex, age at surgery in days), anthropometric (weight (kg), height (cm), BMI (kg/m^2^), and anatomical/hemodynamic parameters (pulmonary annulus diameter (cm and Z-score), maximal instantaneous right ventricular outflow tract (RVOT) gradient (mmHg), baseline peripheral pulse oximetry (%), TOF severity, presence of mitral regurgitation, presence of tricuspid regurgitation, presence of aberrant coronary artery, presence of interatrial shunt, presence of persistent ductus arteriosus, presence of major aortopulmonary collateral arteries (MAPCAs), presence of anomaly of the systemic venous return, and presence of right aortic arch).

Country of origin was included a priori as a potential proxy for structural or systemic factors influencing patient trajectories, such as differences in healthcare access, clinical severity at presentation, or referral patterns. However, due to concerns regarding fairness, transportability, and generalizability, the primary decision tree model was developed excluding country of origin. A secondary model including this variable was evaluated as a sensitivity analysis.

The decision tree model was developed using CRT algorithm in Statistical Package for the Social Sciences (SPSS) software, version 27. We selected the CRT algorithm for its capacity to handle both continuous and categorical variables, its high clinical interpretability, and its in-built pruning mechanisms to prevent overfitting [9, 10].

Model parameters included 10-fold cross-validation, a maximum of tree depth of 5, minimum 20 cases per parent node and 10 per child node, Gini impurity for node splitting, and automatic pruning. Model performance metrics, including overall accuracy, sensitivity, and specificity, are reported together with 95% confidence intervals, which were estimated using bootstrap resampling with 1000 iterations. These metrics are reported for the primary model and for the sensitivity analysis including country of origin.

The primary decision tree model was developed excluding the variable “country of origin”. To assess the robustness of the findings and the contribution of contextual factors, a secondary sensitivity analysis was performed including in the predictor set, while keeping all other parameters unchanged.

For each model, the relative importance of predictor variables was assessed based on their contribution to node splits. Model performance was evaluated using cross-validated estimates of classification error, sensitivity, specificity, and overall accuracy.

To explore potential performance imbalances across patient subgroups, exploratory subgroup error analyses were conducted for the primary model according to sex, age group, and geographic region. Model accuracy and sensitivity were calculated within each subgroup. Given the limited sample size, these analyses were considered descriptive and hypothesis-generating rather than confirmatory.

Ethical approval and data handling procedures were previously described in the original publication [6].

## Results

The baseline demographic, anthropometric and anatomical characteristics of the 115 included patients are summarized in Table 1. Median age at surgery was 1444 days (IQR: 942–2337), with 60% males. Patients originated from 16 different countries across Africa, the Middle East, and Eastern Europe, reflecting the humanitarian referral context.

**Table 1:** Characteristics of the patients

	Total
	(*n* = 115)
Gender	
Male	69 (60.0%)
Female	46 (40.0%)
Country of origin	
Algeria	6 (5.2%)
Burkina Faso	1 (0.9%)
Benin	19 (16.5%)
Cameroun	1 (0.9%)
Chad	1 (0.9%)
Côte d’Ivoire	1 (0.9%)
Djibouti	1 (0.9%)
Georgia	1 (0.9%)
Guinea	12 (10.4%)
Maroc	15 (13.0%)
Mali	13 (11.3%)
Mauritania	15 (13.0%)
Niger	1 (0.9%)
Poland	2 (1.7%)
Senegal	13 (11.3%)
Syria	1 (0.9%)
Togo	5 (4.3%)
Tunisia	7 (6.1%)
Age at surgical repair (days)	1444.00 (942.00 to 2337.00)
Weight (Kg)	12.20 (10.00 to 16.50)
Height (cm)	97.00 (85.00 to 115.00)
BMI (Kg/m^2^)	13.45 (12.49 to 14.71)
TOF Severity	
Mild	38 (33.0%)
Moderate	22 (19.1%)
Severe	55 (47.8%)
Cardiac Associated malformations	
Mitral Regurgitation	
No	107 (93.0%)
Yes	8 (7.0%)
Tricuspid Regurgitation	
No	95 (82.6%)
Yes	20 (17.4%)
Aberrant CA	
No	109 (95.6%)
Yes	5 (4.4%)
Interatrial Shunt	
No	26 (22.6%)
Yes	89 (77.4%)
PDA	
No	63 (55.3%)
Yes	51 (44.7%)
MAPCAs	
No	90 (86.5%)
Yes	14 (13.5%)
ASVR	
No	108 (93.9%)
Yes	7 (6.1%)
RAA	
No	97 (85.1%)
Yes	17 (14.9%)

*PV-SR* Pulmonary Valve - Sparing Repair; *BMI* Body Mass Index; *TOF* Tetralogy of Fallot; *CA* Coronary Artery; *PDA* Persistent Ductus Arteriosus; *MAPCAs* Major Aortopulmonary Collateral Arteries; *ASVR* Anomaly of the Systemic Venous Return; *RAA* Right Aortic Arch; *Kg* kilograms; *cm* centimeter; *m*^*2*^ square meter; *%* percent; *g* grams

A CRT model was generated using data from 115 pediatric humanitarian patients with TOF.

**Fig. 1:**
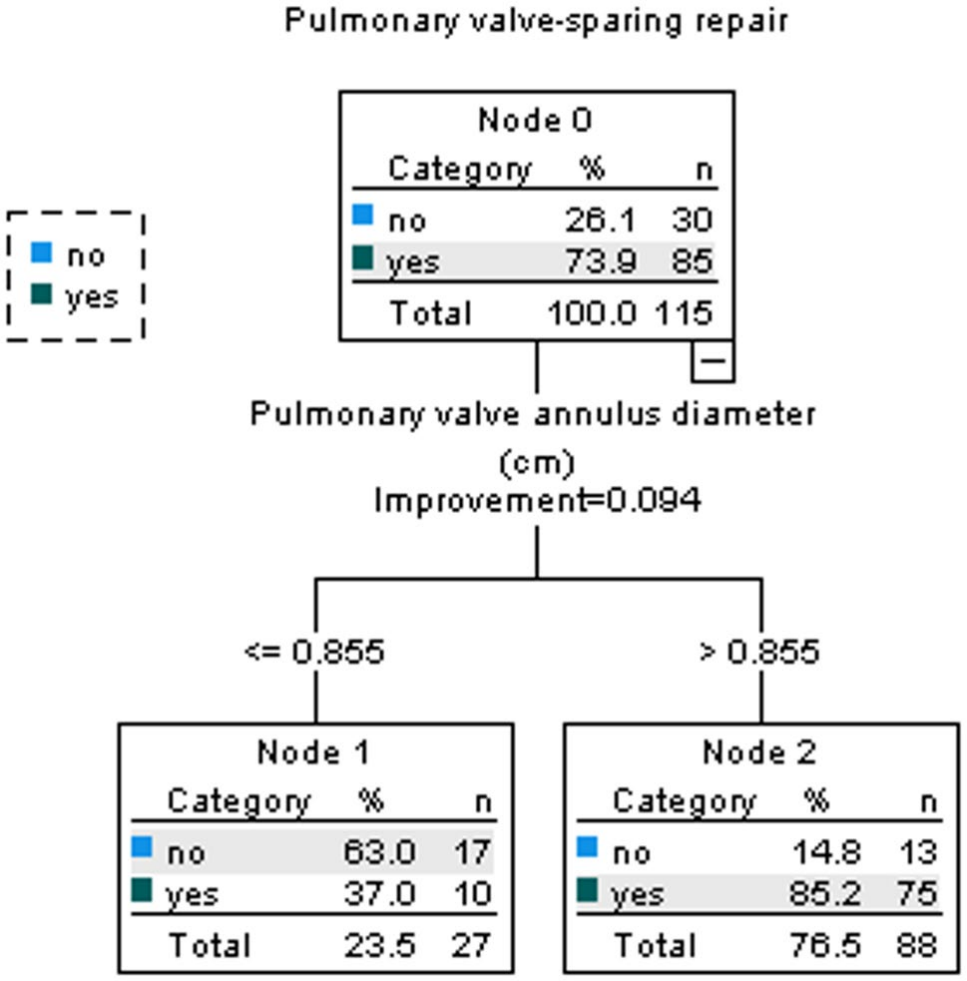
Decision Tree Model for predicting PV-SR in humanitarian pediatric TOF patients. The model uses preoperative variables to stratify patients based on pulmonary valve annulus diameter

The primary decision tree model, developed without including the variable “country of origin”, consisted of a single splitting node based on pulmonary valve annulus diameter. Patients with an annulus diameter > 0.855 cm were classified as likely to undergo PV-SR (Fig. 1).

This model demonstrated an overall accuracy of 80.0% (95% CI:0.72–0.86), sensitivity of 88.2% (95% CI: 0.80–0.94), and specificity of 56.7% (95% CI:0.39–0.73).

**Fig. 2:**
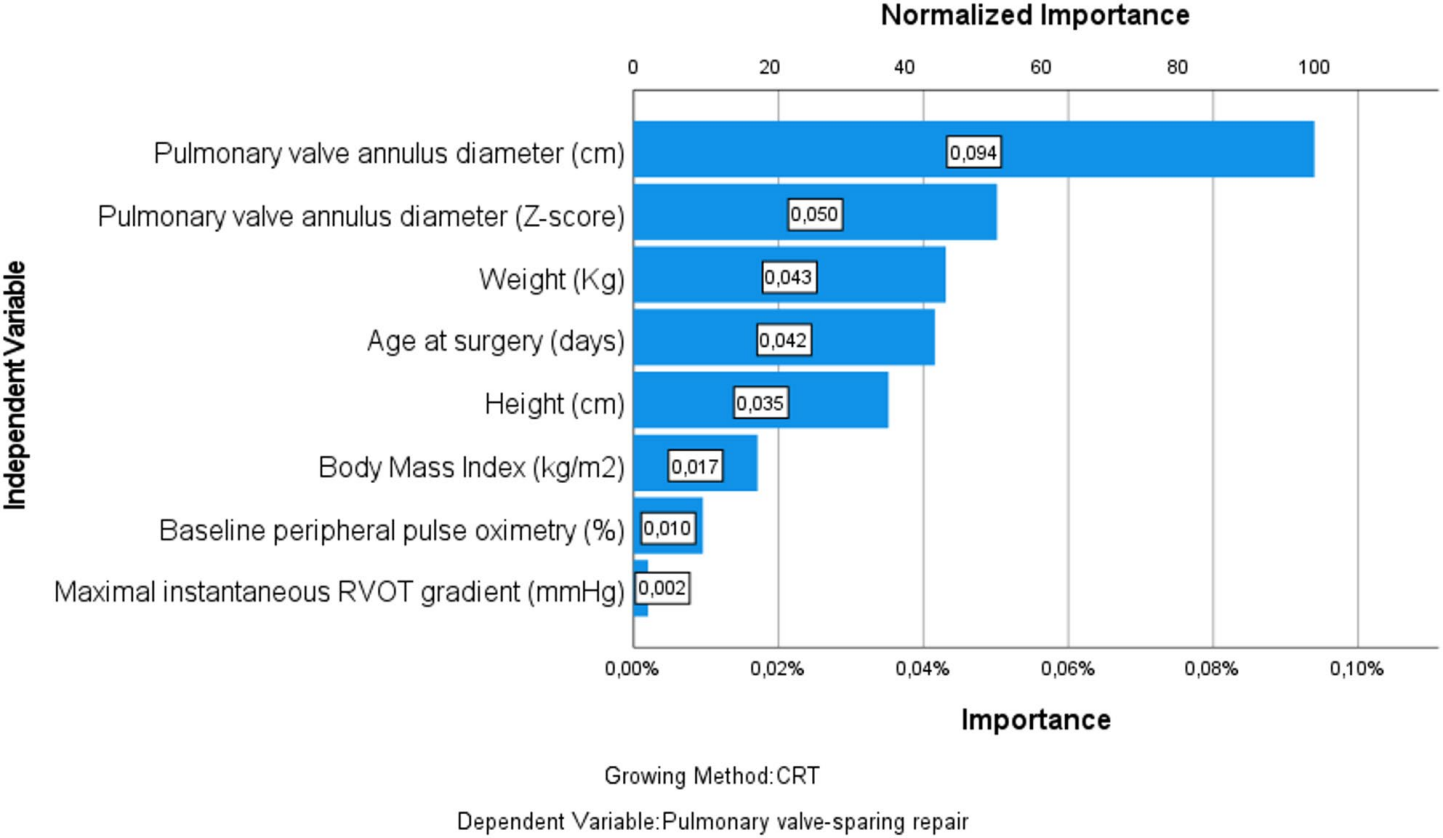
Normalized Variable Importance in the Decision Tree Model for predicting feasibility of PV-SR

The variable importance analysis ranked the pulmonary valve annulus diameter as the most influential predictor (100%), followed by pulmonary annulus Z-score (53.2%). Other variables with moderate importance included weight (45.7%), and age at surgery (44.7%). Variables such as baseline peripheral pulse oximetry (10.6%) and maximal instantaneous RVOT gradient (2.1%) contributed minimally. Several anatomical, including the presence of MAPCAs and mitral regurgitation, were not retained in the final tree structure (Fig. 2; Table 2).

**Table 2:** Raw and normalized importance of predictor variables in the decision tree model

Independent variable	Importance	Normalized importance (%)
Pulmonary valve annulus diameter (cm)	0.094	100
Pulmonary valve annulus diameter (Z-Score)	0.050	53.2
Weight (Kg)	0.043	45.7
Age at surgery	0.042	44.7
BMI (Kg/m^2^)	0.017	18.1
Baseline peripheral pulse oximetry (%)	0.010	10.6
Maximal instantaneous RVOT gradient (mmHg)	0.002	2.1

*cm* centimeter; *Kg* kilograms; *m*^*2*^ square meter; *%* percent; *mmHg* millimeters of mercury

The confusion matrix (Table 3) showed that among the 85 patients who underwent PV-SR, 75 were correctly classified. Among the 30 patients who did not undergo PV-SR, 17 were correctly identified by the model.

**Table 3:** Confusion matrix and performance metrics of the decision tree model

PV-SR	Predicted		
	No	Yes	Total Observed
Observed			
No	17	13	30
Yes	10	75	85
Total Predicted	27	88	115
**Performance Metrics**: Accuracy: 80.0% Sensitivity: 88.2% Specificity: 56.7%	**% (95CI)**		
	80.0 (0.72–0.86)		
	88.2 (0.80–0.94)		
	56.7 (0.39–0.73)		

*PV-SR* Pulmonary Valve - Sparing Repair; *%* percent; *95CI* 95% confidence interval

The supplementary analysis including “country of origin” in the predictor set. The resulting decision tree had two levels: pulmonary valve annulus diameter remained the primary discriminator, followed by country of origin for patients below the threshold (0.855 cm). This model had an overall accuracy of 85.2% (95% CI: 0.78–0.92), sensitivity of 98.8% (95%CI: 0.97–1.00.97.00), and specificity of 46.7% (95%CI: 0.29–0.65). Pulmonary valve annulus diameter was the most influential variable (100% normalized importance), followed by country of origin (76.3%) and annulus Z-score (71.0%) (supplementary material- Fig. 1S and Tables 1 and 2S).

## Discussion

In our previous study, using multivariate logistic regression, we identified pulmonary valve annulus size and BMI as independent predictors of PV-SR in pediatric humanitarian patients with TOF [6].

These patients, originating from LMICs but treated in a high-resource center, represent a distinct population within the broader field of congenital cardiac surgery. International humanitarian surgical programs face unique challenges, including late presentation, lack of local follow-up, and heterogeneity in referral pathways [11, 12].

While informative, traditional logistic regression models assume linearity and additivity among predictors, which may limit their ability to capture complex nonlinear interactions [13]. In this post-hoc analysis, we applied a CRT model to explore whether a decision tree-based approach could provide a clinically intuitive, rule-based tool to support surgical planning.

The decision tree achieved high overall accuracy (80.0%), with sensitivity (88.2%) and specificity (56.7%). This high sensitivity implies a low false-negative rate, which is particularly relevant in humanitarian surgery, where avoiding missed opportunities for PV-SR may reduce the burden of future reoperations and long-term pulmonary insufficiency [14]. Conversely, the lower specificity indicates that some patients predicted to be suitable for PV-SR may ultimately require transannular patching or RV-PA conduit. This overclassification may be acceptable in contexts where the model serves as a preoperative triage tool, rather than a definitive classifier.

However, the clinical implications of false positives – cases predicted as suitable for PV-SR but ultimately requiring alternative surgical approaches – must be carefully considered, particularly in resource-limited humanitarian settings. Although PV-SR generally involves less extensive surgical reconstruction and better long-term outcomes, unnecessary preparation for this technique may still impact operative planning and resource allocation. Thus, while minimizing missed opportunities for PV-SR remains a priority, balanced clinical judgment and intraoperative assessment are essential to confirm surgical feasibility.

Although the final decision to perform PV-SR is made intraoperatively – based on direct inspection of the pulmonary valve, intraoperative Hegar-measurements, and immediate hemodynamic assessment – there remains substantial value in a robust preoperative predictive tool. In humanitarian surgical programs, such models can enhance case triage, support operative planning, standardize decision-making among heterogeneous or rotating teams, and guide counselling for families who frequently arrive with limited diagnostic information. Thus, while not a substitute for intraoperative judgement, a preoperative model serves as an important complementary instrument within the broader continuum of care.

The pulmonary valve annulus diameter (in centimeters), was the most important predictor in the model (100% normalized importance), reaffirming its role as a cornerstone of surgical decision-making in TOF repair. Despite the inclusion of multiple clinical and anatomical preoperative parameters, this single variable alone was sufficient to stratify a large proportion of patients. This is consistent with existing literature that positions annular size as a critical threshold parameter for PV-SR feasibility [3, 6].

Although the CRT identified absolute pulmonary annulus diameter as the primary discriminator, this reflects both anatomical and patient-size considerations. Intraoperatively, surgeons select the Hegar dilator according to the patient’s BSA, aiming to achieve an RVOT dimension with a Z-score of approximately 0. Thus, the absolute diameter integrates the expected annular size and the technical feasibility of PV-SR in a clinically actionable way. In our cohort, absolute diameter and Z-score were strongly correlated, but the CRT algorithm selected absolute diameter for the top split due to slightly higher discriminatory power. Z-score remains an important predictor, providing complementary context. Using absolute diameter as the first split provides a clear and intuitive threshold for surgical planning, particularly in heterogeneous humanitarian patient populations.

In a supplementary analysis including country of origin as a predictor, this variable emerged as the second most influential discriminator for patients with smaller annular diameters. While not a physiological factor, this variable likely captures systemic and contextual influences on patient selection, disease trajectory, and access to care.

Countries grouped under the branch with lower PV-SR rates included Georgia, Algeria, Guinea, Mali, Poland, Senegal, and Morocco. Conversely, countries with high PV-SR rates included Mauritania, Syria, Benin, Tunisia and Togo.

One plausible explanation involves the presence or absence of local pediatric cardiac surgery infrastructure. Countries like Poland, Georgia, Senegal possess established cardiac centers [15, 16]. In these settings, patients with favorable anatomy for PV-SR may be treated locally, while more complex or advanced cases, those less likely to be eligible for PV-SR, are referred abroad. On the other hand, countries like Mauritania, Benin, and Togo with limited access to cardiac surgical care, may tend to refer more stable and less severe cases, which in turn are more likely to be suitable for PV-SR [16].

Importantly, the appearance of “country of origin” as a predictive variable should not be interpreted as reflecting ethnic, racial, or biological differences in RVOT morphology or PV-SR feasibility. Rather, it likely captures upstream structural factors inherent to humanitarian referral systems, including variability in timing of diagnosis, local screening and triage processes, availability of pediatric cardiac surgery, and the types of cases each country refers abroad. In this context, the discriminatory power of this variable reflects differences in patient selection and health-system pathways, rather than intrinsic anatomical predispositions [17, 18].

This finding highlights the importance of considering structural and socioeconomic determinants when analyzing surgical outcomes in global health programs.

Even when surgical treatment occurs in a highly resourced center, the therapeutic pathway is often shaped by pre-admission factors such as referral systems, diagnostic disparities, and logistical barriers, all key components of the continuum of care in humanitarian settings.

The model’s parsimony – reaching only two levels of depth and three terminal nodes – is notable. Despite the breadth of preoperative variables, only a few had discriminative value. Variables such as the Z-score of the pulmonary valve annulus, age at surgery, weight, height, and BMI showed moderate or minimal predictive utility. Body size adjusted measures (e.g. Z-score) may still complement decision-making, but in this model, the absolute diameter had stronger standalone value.

Surprisingly, several expected anatomical and hemodynamic features, including the presence of MAPCs, persistent ductus arteriosus, tricuspid regurgitation, baseline peripheral oxygenation and maximal instantaneous RVOT gradient, did not contribute meaningfully to the classification. These findings may reflect either true lack of predictive value or redundancy/collinearity with dominant variables like annular diameter.

From a methodological standpoint, the CRT approach offers clinical interpretability superior to more complex black-box models [19]. In humanitarian contexts – including both high-income centers treating patients from underserved regions and mission-based care - transparent, rule-based tools may enhance preoperative planning, particularly when multidisciplinary teams rotate and longitudinal data may be limited.

This study has several limitations. It is a single-center retrospective analysis, conducted in a high-resource environment, and may not generalize to all humanitarian programs. The binary outcome (PV-SR vs. no PV-SR) simplifies a nuanced intraoperative process that can be influenced by surgical team experience, intraoperative findings, and evolving strategies. In addition, the retrospective nature of the study limited access to certain preoperative echocardiographic variables, including pulmonary artery branch diameters (LPA and RPA), which were not available in the original dataset. A more detailed assessment of right ventricular function and outflow tract morphology would likely have strengthened the model, as these markers provide important anatomical and prognostic information in TOF repair.

Additionally, only complete cases were analysed, and no multiple-imputation strategy was applied, as missingness mainly affected key anatomical variables and the limited sample size could compromise imputation reliability.

In addition, although we report accuracy, sensitivity, and specificity with 95% confidence intervals to quantify model uncertainty, more extensive internal validation strategies, such as cross-validation across multiple folds, were not performed due to the limited sample size. Formal calibration assessment, including calibration plots and Brier score, was also not undertaken, as reliable estimation would be constrained by the small cohort. Similarly, decision-curve analysis was not performed, given the exploratory nature of the study and the intended role of the model as a preoperative triage and planning aid rather than a definitive decision rule. These limitations are acknowledged and should be addressed in future studies with larger, prospective cohorts.

Moreover, while the inclusion of “country of origin” in the supplementary model reflects real-world referral dynamics, its use in predictive models may raise ethical and methodological considerations regarding equity and potential bias [20]. Similar concerns have been raised in broader machine learning applications in healthcare, where socio-demographic proxies can inadvertently reinforce systemic disparities [20]. To address this, we tested a simplified model excluding this variable, which still achieved good predictive performance (accuracy 80.0%; sensitivity 88.2%; specificity 56.7%). This suggests that equitable and interpretable models are achievable even without potentially bias-inducing contextual variables.

These findings are consistent with our previously published logistic regression model, which identified pulmonary annulus diameter as a strong independent predictor of PV-SR. In that analysis, a receiver operating characteristic (ROC) curve evaluation determined an optimal cutoff of 0.855 cm, yielding a sensitivity of 87.2%, specificity of 61.9%, and an area under curve (AUC) of 0.793 (95% CI: 0.677–0.909; *p* < 0.001) [6].

Our simplified CRT model, excluding the “country of origin” variable, demonstrated comparable predictive performance, with an accuracy of 80.0%, sensitivity of 88.2%, and specificity of 56.7%. This suggests that the decision tree approach can achieve robust and interpretable prediction without relying on potentially bias-inducing socioeconomic proxies.

This aligns with recent literature on fairness in machine learning, which advocates for approaches such as “fairness through unawareness” – developing models that intentionally exclude sensitive or proxy attributes – and model audits to identify potential disparities in predictions across subgroups [21, 22]. Applying these principles in humanitarian surgical contexts is particularly relevant, as decisions based on biased models could inadvertently reinforce global health inequities. Our findings support the feasibility of ethical, equitable predictive tools without compromising clinical utility.

While accuracy and AUC are not identical metrics, their comparable values indicate overlapping model performance. Furthermore, the CRT model offers enhanced interpretability through a hierarchical, rule-based structure, which can be advantageous in clinical decision-making, particularly in humanitarian surgical settings where minimizing false negatives is critical.

Future work should include external validation across multiple humanitarian programs, including those operating directly in LMICs [23, 24]. The development of a web- or app-based interface could enhance usability, and ensemble learning techniques could be explored to improve specificity while preserving interpretability. More importantly, ongoing assessment of how such tools influence surgical outcomes, resource allocation, and equity of access will be critical.

## Conclusion

The decision tree model, applied to a humanitarian cohort of pediatric TOF patients, identified the pulmonary valve annulus diameter (in centimeters) the primary predictor of PV-SR feasibility. Despite expanding the clinical dataset, the model retained a simple, robust, and highly interpretable structure, offering 85.2% accuracy with excellent sensitivity.

Importantly, the influence of non-anatomical factors such as country of origin – even in a high-standard surgical environment- highlights the need to account for upstream systemic disparities in global health planning. Structural barriers, selection biases, and differential access to care must be acknowledged as part of the surgical care continuum.

This model may offer a useful preoperative triage tool within humanitarian cardiac surgery programs, supporting more transparent, equitable, and efficient decision-making across diverse global settings. In practical terms, this decision tree model could be useful in preoperative planning or team discussions to quickly estimate the feasibility of PV-SR. It may also aid referral triage in humanitarian programs, helping prioritize patients and optimize resources. Future work could focus on developing this model into an accessible digital tool for wider clinical use. External validation in other humanitarian cohorts and geographical contexts is essential before clinical adoption.

## Supplementary Information

Supplementary Material 1

Supplementary Material 2

## Data Availability

Data is provided within the manuscript or supplementary information files.
