# Supplementary material for: Preoperative decision tree model for predicting pulmonary valve-sparing repair in humanitarian pediatric tetralogy of fallot patients
Source: J Cardiothorac Surg. 2026 Mar 2;21:161. doi: 10.1186/s13019-026-03882-8 (PMC13059338; doi:10.1186/s13019-026-03882-8)
Supplement: Supplementary file 1 — Supplementary Material 1 [file 13019_2026_3882_MOESM1_ESM.docx]

**Supplementary Material**

~~
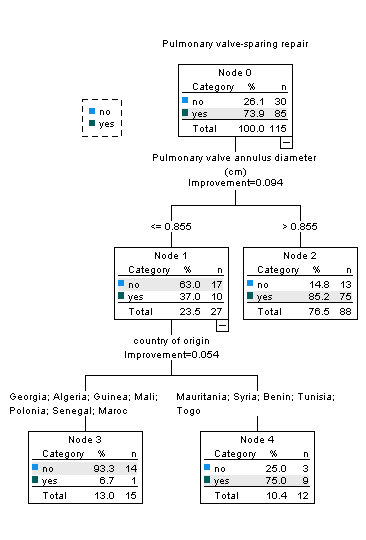
~~

**Figure 1S: Decision Tree Model for predicting PV-SR in humanitarian pediatric TOF patients. The model uses preoperative variables to stratify patients based on pulmonary valve annulus diameter**


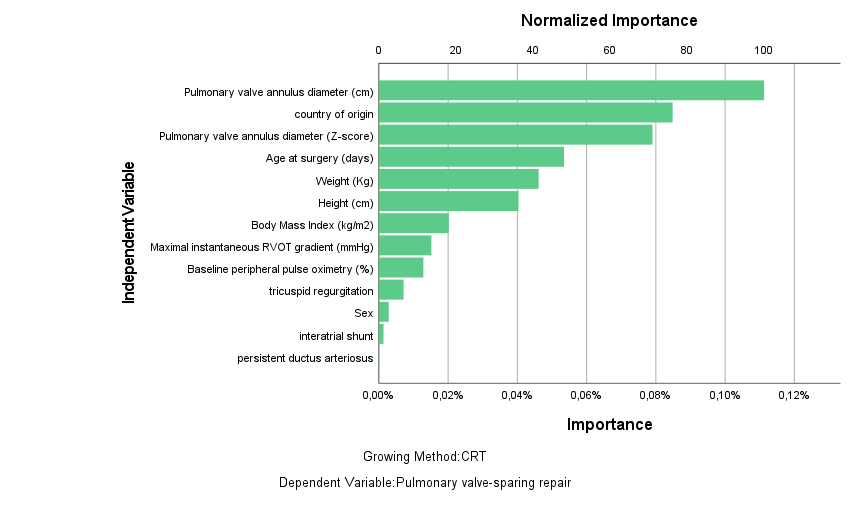


**Figure 2S: Normalized Variable Importance in the Decision Tree Model for predicting feasibility of PV-SR.**

The variable importance analysis ranked the pulmonary valve annulus diameter as the most influential predictor (100%), followed by country of origin (76.3%) and pulmonary annulus Z-score (71.0%). Other variables with moderate importance included age at surgery (48.1%), weight (41.5%), and height (36.3%). Variables such as tricuspid regurgitation (6.4%), sex (2.6%), interatrial shunt (1.2%) and persistent ductus arteriosus (0.1%) contributed minimally. Several anatomical and hemodynamic parameters, including the presence of MAPCAs and mitral regurgitation, were not retained in the final tree structure.

**Table 1S: Raw and Normalized Importance of Predictor Variables in the Decision Tree Model**

| **Independent Variable** | ***Importance*** | ***Normalized Importance (%)*** |
| --- | --- | --- |
| Pulmonary valve annulus diameter (cm) | 0.111 | 100 |
| Country of origin | 0.085 | 76.3 |
| Pulmonary valve annulus diameter (Z-Score) | 0.079 | 71.0 |
| Age at surgery | 0.053 | 48.1 |
| Weight (Kg) | 0.046 | 41.5 |
| Height (cm) | 0.040 | 36.3 |
| BMI (Kg/m^2^) | 0.020 | 18.2 |
| Maximal instantaneous RVOT gradient (mmHg) | 0.015 | 13.6 |
| Baseline peripheral pulse oximetry (%) | 0.013 | 11.6 |
| Tricuspid regurgitation | 0.007 | 6.4 |
| Sex | 0.003 | 2.6 |
| Interatrial shunt | 0.001 | 1.2 |
| Persistent ductus arteriosus | 8.94x10^-5^ | 0.1 |

*Abbreviations: cm* centimeter; *Kg* kilograms; *m^2^* meters quarter; *mmHg* millimeters of mercury; *%* percent

**Table 2S: Confusion Matrix and Performance Metrics of the Decision Tree Model**

| *PV-SR* | *Predicted* | | |
| --- | --- | --- | --- |
|  | *No* | *Yes* | *Total Observed* |
| Observed |  |  |  |
| No | 14 | 16 | 30 |
| Yes | 1 | 84 | 85 |
| Total Predicted | 15 | 100 | 115 |
| **Performance Metrics**  Accuracy  Sensitivity  Specificity | **% (95CI)** |  |  |
|  | 85.2 (0.78-0.92) |  |  |
|  | 98.8 (0.97-1.00) |  |  |
|  | 46.7 (0.29-0.65) |  |  |

*Abbreviations: PV-SR* Pulmonary Valve - Sparing Repair; *%* percent; *95CI* 95% confidence interval
